# The Effect of Outer Space and Other Environmental Cues on Bacterial Conjugation

**DOI:** 10.1128/spectrum.03688-22

**Published:** 2023-03-30

**Authors:** Bar Piscon, Eliana Pia Esposito, Boris Fichtman, Guy Samburski, Lihi Efremushkin, Shimon Amselem, Amnon Harel, Galia Rahav, Raffaele Zarrilli, Ohad Gal-Mor

**Affiliations:** a The Infectious Diseases Research Laboratory, Sheba Medical Center, Tel-Hashomer, Israel; b Department of Clinical Microbiology and Immunology, Tel Aviv University, Tel Aviv, Israel; c Sackler Faculty of Medicine, Tel Aviv University, Tel Aviv, Israel; d Department of Public Health, University of Naples Federico II, Naples, Italy; e Azrieli Faculty of Medicine, Bar-Ilan University, Safed, Israel; f SpacePharma R&D Israel LTD., Herzliya Pituach, Israel & SpacePharma SA, Courgenay, Switzerland; University of Nebraska-Lincoln

**Keywords:** HGT, T4SS, conjugation, conjugative pili, environmental cues, Gram-negative bacteria, microgravity, outer space, plasmid, tra genes

## Abstract

Bacterial conjugation is one of the most abundant horizontal gene transfer (HGT) mechanisms, playing a fundamental role in prokaryote evolution. A better understanding of bacterial conjugation and its cross talk with the environment is needed for a more complete understanding of HGT mechanisms and to fight the dissemination of malicious genes between bacteria. Here, we studied the effect of outer space, microgravity, and additional key environmental cues on transfer (*tra)* gene expression and conjugation efficiency, using the under studied broad-host range plasmid pN3, as a model. High resolution scanning electron microscopy revealed the morphology of the pN3 conjugative pili and mating pair formation during conjugation. Using a nanosatellite carrying a miniaturized lab, we studied pN3 conjugation in outer space, and used qRT-PCR, Western blotting and mating assays to determine the effect of ground physicochemical parameters on *tra* gene expression and conjugation. We showed for the first time that bacterial conjugation can occur in outer space and on the ground, under microgravity-simulated conditions. Furthermore, we demonstrated that microgravity, liquid media, elevated temperature, nutrient depletion, high osmolarity and low oxygen significantly reduce pN3 conjugation. Interestingly, under some of these conditions we observed an inverse correlation between *tra* gene transcription and conjugation frequency and found that induction of at least *traK* and *traL* can negatively affect pN3 conjugation frequency in a dose-dependent manner. Collectively, these results uncover pN3 regulation by various environmental cues and highlight the diversity of conjugation systems and the different ways in which they may be regulated in response to abiotic signals.

**IMPORTANCE** Bacterial conjugation is a highly ubiquitous and promiscuous process, by which a donor bacterium transfers a large portion of genetic material to a recipient cell. This mechanism of horizontal gene transfer plays an important role in bacterial evolution and in the ability of bacteria to acquire resistance to antimicrobial drugs and disinfectants. Bacterial conjugation is a complex and energy-consuming process, that is tightly regulated and largely affected by various environmental signals sensed by the bacterial cell. Comprehensive knowledge about bacterial conjugation and the ways it is affected by environmental cues is required to better understand bacterial ecology and evolution and to find new effective ways to counteract the threating dissemination of antibiotic resistance genes between bacterial populations. Moreover, characterizing this process under stress or suboptimal growth conditions such as elevated temperatures, high salinity or in the outer space, may provide insights relevant to future habitat environmental conditions.

## INTRODUCTION

Horizontal gene transfer (HGT) is a powerful force in prokaryote evolution, in which foreign DNA from a distinct (and often distantly) genetic origin is being delivered and assimilated in the genome of the accepting bacterial cell (1, 2). Today, three canonical HGT mechanisms, termed transformation, transduction, and conjugation (3, 4) and a fourth mode of intercellular DNA transfer called vesiduction, which involves secretion and uptake of extracellular vesicles (5), are known. From all HGT mechanisms characterized so far, conjugation is the most common route for the dissemination of resistance and virulence genes among bacteria (6, 7). The process of conjugation involves the transfer of DNA in a unidirectional manner from a donor bacterium to a recipient cell by transenvelope channel structures, through which DNA can pass. The conjugative systems with the highest complexity are those encoded by Gram-negative bacteria either by relatively large, self-transmissible plasmids or by chromosomally integrated mobile genetic units called integrated conjugative elements (ICEs), that can be excised and move between cells as a plasmid intermediate (8).

In Gram-negative bacteria, these conjugative transfer systems involve several functional elements, including a DNA-processing complex termed “relaxosome”; a large membrane-embedded machinery termed conjugative type IV secretion system (T4SS); and an extracellular conjugative pilus (9). The genes that encode these elements are called transfer *(tra*) genes and they are often clustered together in the *tra* region of conjugative plasmids.

In Gram-negative bacteria, a minimum of 12 proteins (VirB1 to VirB11 and VirD4) compose the T4SS responsible for pilus biogenesis and transferring the conjugation cargo (10). The genetic framework of these systems is organized into two functional groups defining the DNA transfer and replication (Dtr) module and the mating pair formation (Mpf) system, which is required for the production of the conjugative pili and the formation of cell-to-cell junctions, used for the translocation of the DNA-protein substrates. A third function, interceding between Dtr and Mpf, is fulfilled by the coupling protein (CP, VirD4) (11).

According to the currently accepted model for Gram-negative bacteria, the conjugative pilus extends off the outer membrane of the donor bacterium and establishes contact with a recipient cell. The pilus then retracts, pulling the recipient bacterium toward the donor cell and a tight conjugative junction is formed (9). During conjugation, a single-stranded DNA substrate called the T-strand is generated in the donor cell by the relaxase and additional accessory proteins of the relaxosome. The relaxase nicks one strand of the dsDNA at the origin of transfer (*oriT*) loci and initiates a rolling circle type replication. The relaxase together with the other relaxosome-associated proteins remains covalently bound at the 5′ end of the T-strand, while directing this complex via the preassembled T4SS, directly into the recipient cell (12–14). The sequence adjacent to the *oriT* is known as the leading region and will be the first to enter the recipient cell as a ssDNA–protein substrate. This region encodes ssDNA binding (SSB) protein, anti-restriction and SOS inhibition proteins, required to protect the T-strand from enzymatic degradation to facilitate its conversion into a dsDNA plasmid upon entry into the recipient cell, and for generating stable transconjugants (15).

Bacterial conjugation is a complex and energy-consuming process, which requires sensing and regulatory strategies that ensure accurate *tra* gene expression at the precise time and place. Since the expression of *tra* genes can inflict a significant metabolic burden (fitness cost) and cellular stress (16), their expression is tightly regulated by different plasmid and host factors that act together to repress the conjugation gene expression, under unfavorable conditions. In F-plasmids, the expression of *tra* genes is controlled by transcriptional and posttranscriptional mechanisms, including the plasmid-encoded transcription activator, TraJ and several host-encoded proteins that are sensitive to nutrient availability and stress, including ArcA, Lrp, and H-NS (17). In F-plasmids, the expression of *traJ*, and therefore that of other transfer genes, is posttranscriptionally repressed by the fertility inhibition system FinOP. Consequently, TraJ repression keeps conjugation in the “OFF” state (18) that can be relieved in response to specific environmental and physiological stimuli, as well as by the presence of recipient cells (13, 19). Therefore, quorum sensing and a response to specific environmental cues such as oxygen levels, nutrients, and temperature are generally required to induce *tra* gene expression.

Since their discovery in the late 1940s, F and F-like plasmids have been used as a prototype to study bacterial conjugation and were extensively investigated (20). In contrast, the conjugation systems of broad-host range plasmids such as IncN plasmids are still under studied and less understood. Previous studies have shown that although they share the same functional elements, the conjugal transfer systems and the genetic organization of *tra* genes in IncN plasmids are very different than the one in F plasmids (21). Considering the increasingly apparent diversity of various conjugation systems (22, 23) a better understanding of the regulation of different bacterial conjugation systems and their response to different environmental conditions is needed for a more complete understanding of general principles in conjugation biology and to fight the spread of antibiotic resistance and other malicious genes via conjugation.

pN3 (Accession NC_015599), a broad-host range IncN plasmid, 54,205 bp long, has a GC content of 51.1% and encodes 62 putative ORFs, including the *tetA* gene, conferring tetracycline resistance (24). Here, we sought to study the effect of outer space, microgravity, and additional key environmental cues on *tra* gene expression and bacterial conjugation using the plasmid pN3 as a model of a non-F conjugation plasmid.

## RESULTS

### Imaging pN3-mediated conjugation.

**FIG 1:**
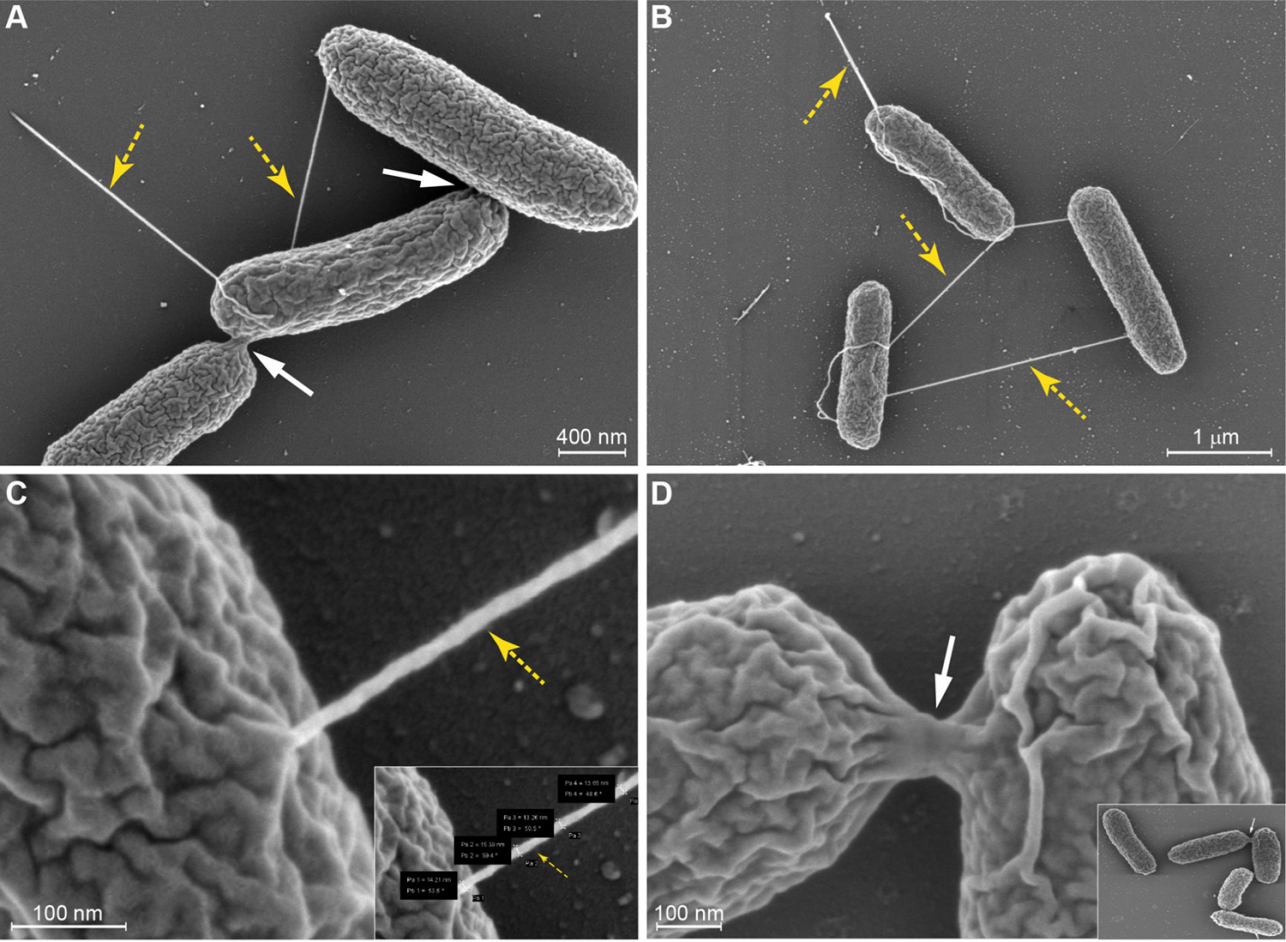
Scanning EM imaging of pN3 conjugative pili and MPFs. E. coli K-12 ORN172 “naked” strain that does not express fimbriae harboring pN3 was grown on selective LB plates. Merlin scanning EM was used to image the cells following iridium coating. (A) Yellow arrows show the conjugative pili and white arrows indicate cell-to-cell contact points and the mating pair formation (MPFs). (B) The morphology of the conjugation pili that were expressed as 1 to 2 pili per cell is shown. (C) The ImageJ measurement tool was used to measure the length (mean 1.89 ± 1.2 μm) and the diameter (mean 14.85 ± 1.89 nm) of the pN3 conjugative pili. (D) An enlarged image of the conjugative bridge formed by two mating cells.

Bacterial conjugation is a contact-dependent process, which involves a conjugative pilus associated with a T4SS. Previous studies that have used electron microscopy imaging approaches were successful in characterizing the morphology of conjugation pili of plasmids belonging to different incompatibility groups. Here, to visually characterize the conjugation pili and cell-to-cell contact formation during conjugation of pN3 we have used an Escherichia coli K-12 “naked” strain ORN172 as a surrogate host. This strain was deleted from all genes encoding fimbriae (25) and was previously used for imaging of xenogeneic pili (26, 27). Using delicate, gain-less iridium coating and high-resolution scanning electron microscopy (SEM) (28, 29), we were able to image in E. coli ORN172 carrying pN3 both the conjugation pili (Fig. 1A to C; yellow arrows) and the conjugation bridges that were formed between mating cells (Fig. 1A and D; white arrows). Current conjugative models suggest that the conjugative pili are extended to capture another bacterium and, if successful, retract in order to bring the two interacting bacteria close together. This forms a stable cell-to-cell contact and facilitates the transfer of a plasmid copy to the recipient, which in turn becomes a transconjugant (8).

In agreement with this view, under our experimental conditions (transconjugants growing on LB agar plates at 37°C), we found 1–2 rigid conjugative pili per cell, that appear to be randomly arranged on the cell surface. These pili appeared shorter, thicker and more rigid than the flagella (Fig. S1) with a tubular structure of 1.89 ± 1.2 μm in length (*n* = 242) and 14.85 ± 1.89 nm in diameter (*n* = 71). Tight cellular junctions that were previously suggested to facilitate high efficiency mating pair formation (30) were imaged as cell-to-cell bridges of about 100 nm width (Fig. 1D). To the best of our knowledge, these are the first published EM images of an IncN plasmid mating pair formation and intact conjugative pili structures.

### Bacterial conjugation occurs in outer space.

Outer space and growth under microgravity conditions have previously been shown to profoundly affect bacterial physiology and gene expression. Bacterial suspension cultures of E. coli, Bacillus subtilis, and *Staphlyococcus aureus* were shown to grow to higher stationary-phase concentrations and had shorter lag-phase during space flight and under a ground-based model of microgravity (31–33). Similarly, enhanced biofilm formation was found in E. coli and Pseudomonas aeruginosa grown under modeled reduced gravity conditions (34, 35). Moreover, space flight was previously shown to affect the expression of *tra* genes in Salmonella enterica serovar Typhimurium plasmids in a Hfq-dependent manner (36).

**FIG 2:**
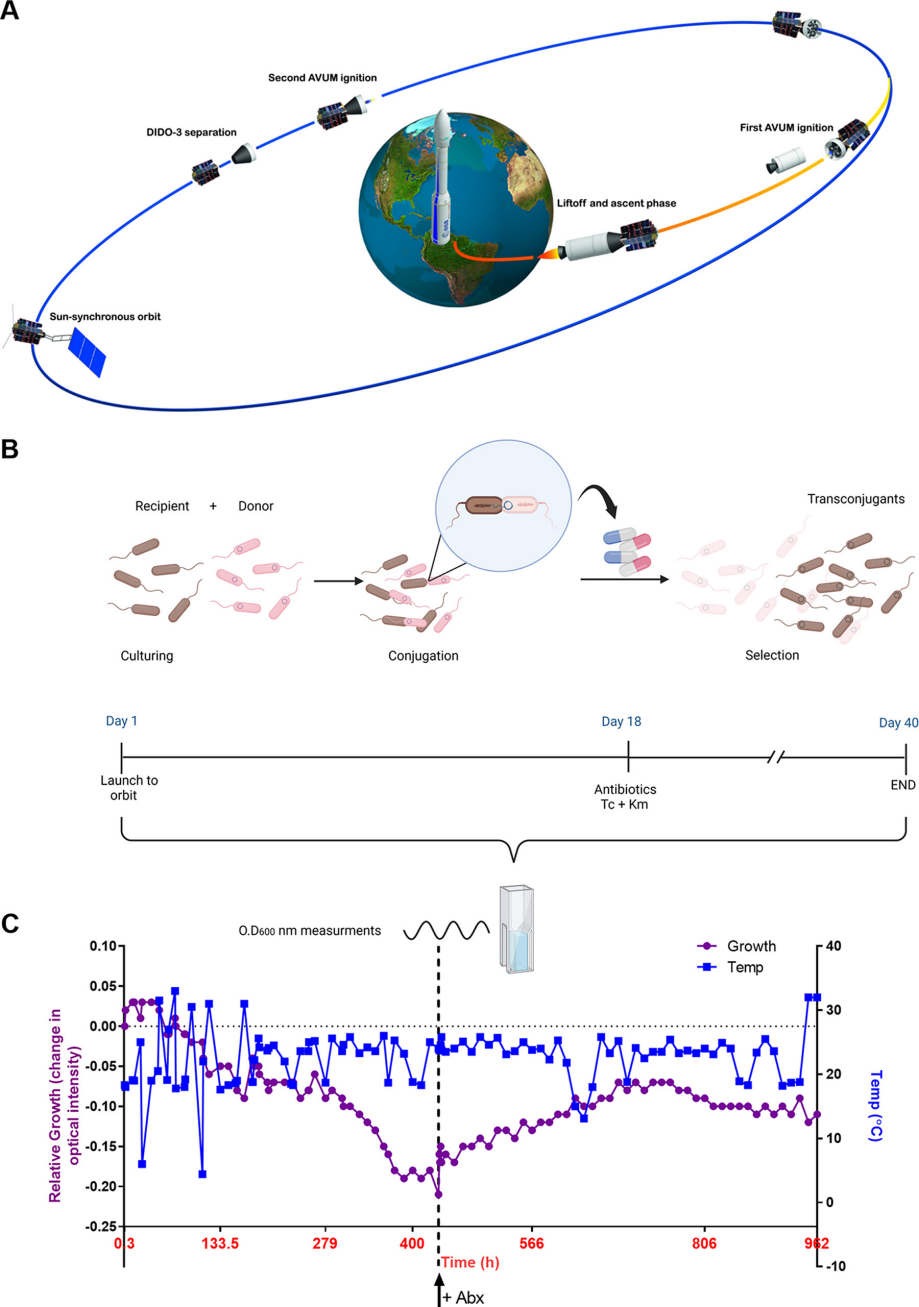
Bacterial conjugation occurs in outer space. (A) Schematic illustration of the experimental setup in outer space. A miniaturized microgravity laboratory platform SPmg2Lab was installed on the DIDO-3 research nanosatellite. DIDO-3 nanosatellite carrying the conjugation experiment was launched from the Guiana Space Centre using a Vega rocket flight number VV16 (Arianespace). One hour and 45 min after launching DIDO-3 was separated from Vega rocket and entered an orbit course altitude of 530 km around earth with an earth orbit time of 90 min. The image was created and modified, with permission, from an illustration made by Arianespace (https://www.arianespace.com/). (B) The experimental setup of the conjugation experiment in outer space. On orbit, E. coli K1037 harboring pN3 as a donor strain and E. coli K-12 ORN172 carrying a chromosomal kanamycin resistance, as a recipient were mixed in a 1:1 ratio in the observation chamber of the SPmg2Lab and maintained in fresh LB without selection for 18 days, allowing conjugation to take place. To select for transconjugants, fresh LB supplemented with tetracycline and kanamycin was added to the observation chamber and the growth of transconjugants was followed for an additional 22 days using optical readings that were performed by an integrated spectrophotometer at 600 nm. (C) The temperature in the observation chamber and the relative growth (change in optical intensity from time zero) of the cultures over 40 days in space is shown. The time of the addition of fresh broth and antibiotics, which were used for transconjugant selection is indicated by a red arrow and a broken line.

Based on these findings, we asked whether bacterial conjugation, that requires precise regulation and the assembly of a multicomponent conjugation apparatus could occur in microgravity and in outer space. To address this question, we utilized a remote controlled autonomous miniaturized microgravity laboratory platform called SPmg2Lab that was developed by SpacePharma (Fig. S2C) (37). This platform was installed on the DIDO-3 nanosatellite equipped with solar cells and batteries for power supply and communication system allowing autonomous remote control of the experiments from earth. The DIDO-3 nanosatellite (Fig. S2A-B), carrying the miniaturized laboratory platform was launched from the Guiana Space Centre (Europe's Spaceport, Kourou, French Guiana) to an orbit altitude of 530 km around earth on September 3, 2020, using a Vega rocket flight number VV16 (Arianespace; Fig. 2A). On orbit, we mixed in fresh LB medium in a 1:1 ratio the E. coli K1037 harboring pN3 as a donor strain and E. coli K-12 ORN172 carrying a chromosomal kanamycin resistance, as a recipient bacterium. The donor and the recipient mixed culture was grown in LB without antibiotics for 18 days, allowing conjugation to occur in space. To select for transconjugants (E. coli ORN172 that have acquired pN3), fresh LB supplemented with tetracycline (to a final concentration of 20 μg/mL) and kanamycin (to a final concentration of 50 μg/mL) was added to the SPmg2Lab observation chamber and the growth of transconjugants (by reading change in optical intensity) was followed for an additional period of 22 days (Fig. 2B). During the entire time of the experiment that lasted 40 days on orbit, the optical intensity of the mixed culture was measured using an integrated spectrophotometer three to four times a day and sent to the control ground station in system communication packages. Fig. 2C shows the growth of the bacterial cultures in space over 40 days before and after antibiotic selection and the measured temperature of the observation chamber that fluctuated between 4.4 to 31.5°C (average temperature was 22.2°C) while on orbit. Noticeably, after the addition of both antibiotics to the mixed cultures, we observed a clear and stable increase in optical intensity (bacterial growth) during the next 13 days. Since multiple ground experiments did not detect a spontaneous rising of a tetracycline- and kanamycin-resistant clones in the recipient or the donor bacteria alone without mating (Table S3), including in cultures that were incubated for 40 days as the space experiment (see Table S4 and supplementary text), we concluded that the increased optical intensity reflected the growth of the newly formed transconjugants resistant to tetracycline and kanamycin. These results indicated that successful pN3 conjugation and dissemination of antibiotic resistance genes can occur on orbit, in outer space.

### pN3 conjugation is affected by microgravity.

**FIG 3:**
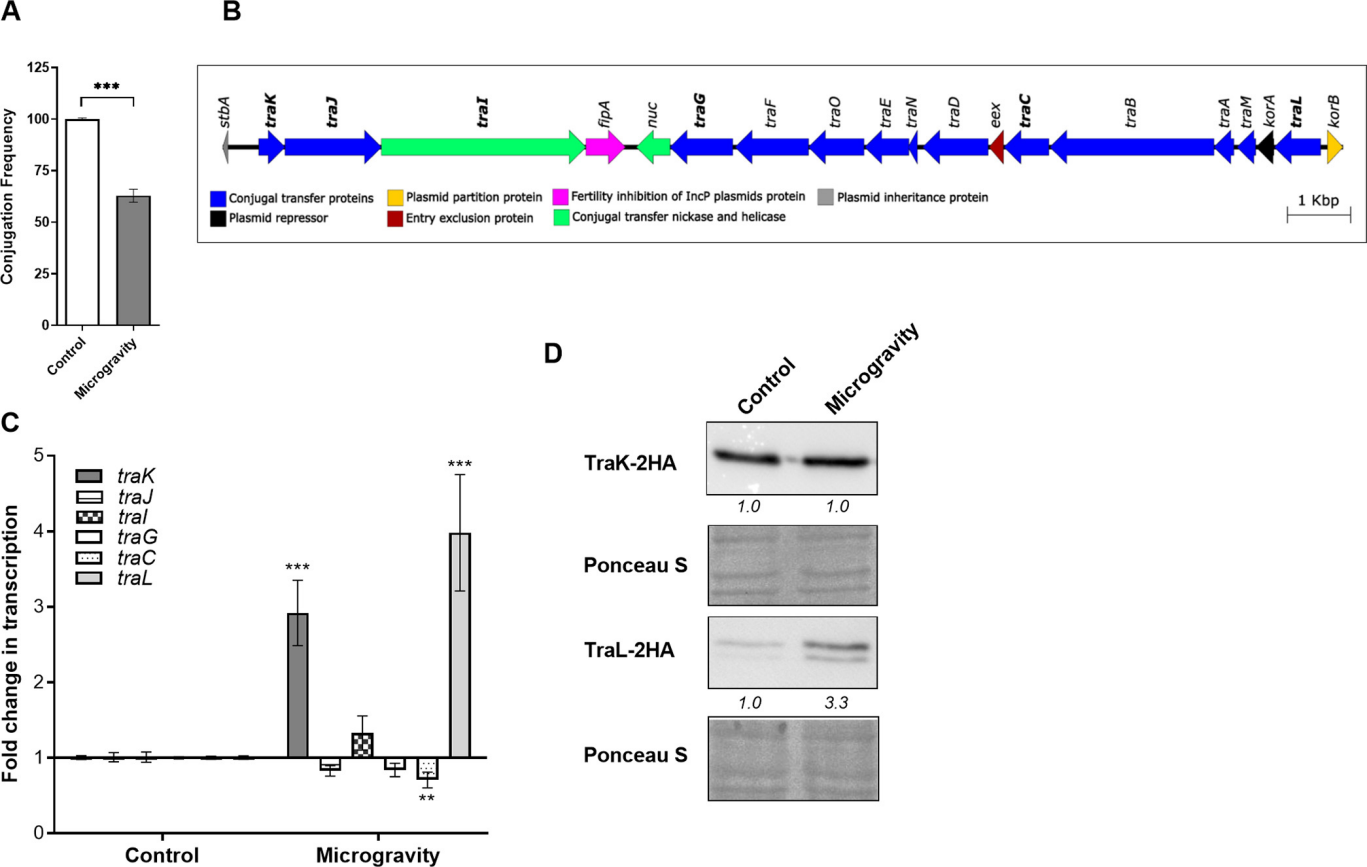
Bacterial conjugation is affected by microgravity. (A) Equal amounts of the donor (E. coli K1037 harboring pN3 plasmid) and the recipient (E. coli K-12 ORN172) strains were mixed in liquid LB medium and placed on the Rotating Wall Vessel (RWV) bioreactor at 37°C for 6 h. An identical control experiment included mixing the same cultures in an RWV disposable vessel and mounting it on a standard roller drum rotating at 43 RPM. Conjugation frequency was determined by the ratio of the obtained transconjugant CFU (resistant to tetracycline and kanamycin) and the initial number of donor CFU. The columns show the mean conjugation frequency of six independent experiments and their SEM. *t* test was used to determine statistical significance. ***, *P* < 0.001. (B) The genetic organization of the *tra* region in pN3 (accession number FR850039 position 9618 to 27789) is shown. The conjugation *tra* genes cluster contains 20 ORFs encoding the conjugation transfer proteins (blue), plasmid partition protein (yellow), fertility inhibition (magenta), plasmid inheritance protein (gray), transcriptional regulator (black), entry exclusion protein (red), and conjugational transfer nickase and helicase (green). (C) RNA was extracted from E. coli K1037 harboring pN3 grown in LB under microgravity-simulated conditions in an RWV bioreactor (microgravity), and from control cultures that were grown simultaneously under gravity conditions (control). The transcription levels of *tra*K, *traJ*, *traI*, *traG*, *traC,* and *traL* was determined by qRT-PCR and normalized to the 16S rRNA gene. The bars represent the mean and SEM of 6–12 reactions from 2–4 independent RNA extractions. One Sample *t* test against a theoretical value of 1.0 was used to determine statistical significance. **, *P* < 0.01; ***, *P* < 0.001. (D) E. coli K1037 harboring pN3 and expressing a 2HA-tagged version of TraL and TraK were grown on RWV bioreactor under microgravity-like conditions and under gravity for 6 h at 37°C. The expression of TraK-2HA and TraL-2HA was determined by Western blotting of whole-cell lysate, using an anti-2HA antibody. The transferred membranes were stained with Ponceau S and imaged for a loading control. Quantified bends densitometry is shown under the blots of TraK-2HA and TraL-2HA.

To further explore the effect of microgravity on bacterial conjugation we used a microgravity ground simulator called the rotating wall vessel (RWV) bioreactor, that was developed by the National Aeronautics and Space Administration (NASA) in the early 1990s. This device is used as a microgravity simulator to mimic and model the effects of microgravity and low fluid-shear environment on cells in earth-based laboratory studies. Cells growing in the RWV bioreactor are subjected to a continuous free fall, while effectively simulating two key aspects of the microgravity culture environment, including a continuous suspension condition and minimized turbulence and shearing forces (35, 37). Interestingly, while bacterial cultures that were grown for 6 h in the RWV bioreactor demonstrated similar viability (CFU count) to cultures grown in the same vessel on a standard roller drum (both instruments were rotated at 43 RPM), the frequency of conjugation in the RWV bioreactor grown cultures was ~40% lower than the conjugation frequency of cultures grown on the roller drum (Fig. 3A), suggesting that microgravity-simulated conditions significantly reduce bacterial conjugation frequency.

To better understand the effect of microgravity-simulated conditions on bacterial conjugation, we next determined changes in the transcription of *tra* genes in E. coli K1037 harboring pN3 grown under microgravity on the RWV bioreactor versus cultures grown under normal gravity on a typical roller drum. To this end, we have chosen to look at the expression of six *tra* genes [*traG* (a VirB11 homologue, encoding a T4SS ATPase (38)), *TraL* (see below), *traK* (see below), *traC* (a VirB5 homologue, encoding a conjugal transfer protein (39)), *traI* (encoding a conjugative relaxase (40)), and *traJ* (encoding a T4SS coupling protein with a DNA-binding domain) as representative genes organized along the transfer region of pN3 (Fig. 3B)]. Interestingly, quantitative real-time reverse transcription (qRT-PCR) indicated that although conjugation frequency decreased, the transcription of *traK* and *traL* was moderately induced by about 3- and 4-fold, respectively, in cultures grown under microgravity conditions in comparison to cultures grown in similar conditions, but under normal gravity (Fig. 3C). TraK is a DNA transfer and replication (Dtr) protein and a predicted member of the Ribbon-Helix-Helix family of DNA-binding proteins, involved in the transfer of DNA and protein substrates through the conjugation systems of IncN plasmids (21, 40, 41). TraL is an Agrobacterium tumefaciens VirB1 orthologue, a putative lytic transglycosylase and is required for the T4SS assembly (42).

To account for changes in the expression of Tra proteins and confirm these results, we have constructed 2- hemagglutinin (2-HA) tagged versions of TraK and TraL, encoded on the opposite sides of the *tra* region, and cloned them under their native promoter in a low-copy number plasmid (pWSK29), that was introduced into E. coli K1037 carrying pN3. In partial agreement with the transcriptional data, Western blotting indicated similar translation levels of TraK-2HA at both conditions, but elevated translation of TraL-2HA under microgravity-simulated conditions compared to cultures grown at gravity (Fig. 3D). We concluded from these experiments that while bacterial conjugation can happen in outer space, microgravity may moderately reduce its frequency, and affect the expression of at least some *tra* genes, both at the transcriptional and translational levels.

### The effect of growth media on pN3 conjugation.

**FIG 4:**
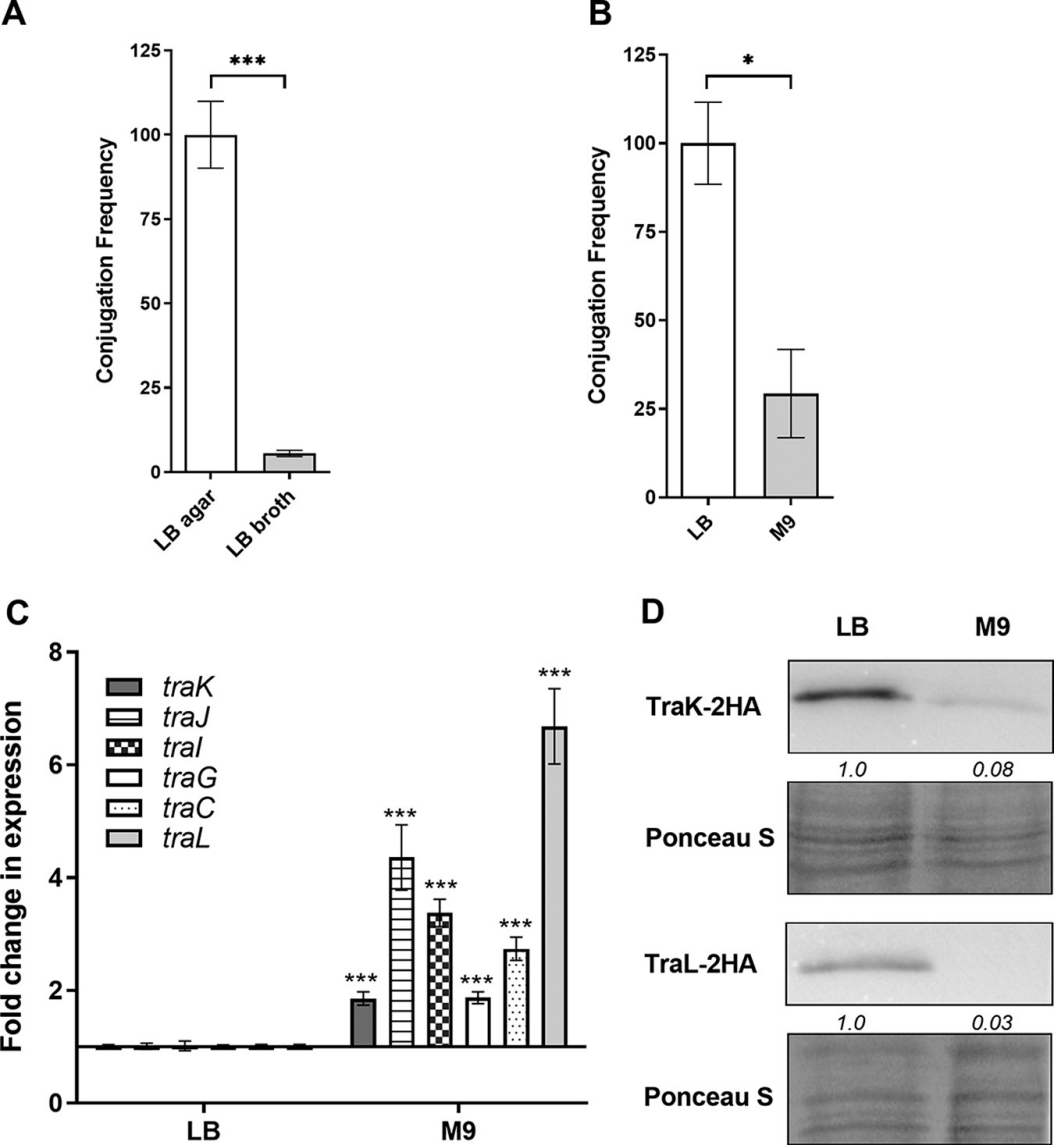
pN3 conjugation is induced on solid and rich medium. (A) Cultures of the donor (E. coli K1037 harboring pN3 plasmid) and the recipient (E. coli K-12 ORN172) strains that were grown overnight in LB washed and resuspended in fresh LB medium. Equal amounts (10 μL) were mixed together and spotted onto LB agar plates. In parallel, 100 μL of the donor and recipient resuspended in liquid LB were mixed together in a test tube. Both mating mixtures were incubated for 6 h at 37°C. pN3 conjugation frequency was determined by CFU enumeration of the obtained transconjugants relative to the initial number of donor CFU, following plating of serial dilutions on selective plates. The chart shows the mean of three independent experiments with the SEM indicated by the error bars. *t* test was used to determine statistical significance. ***, *P* < 0.001. (B) Cultures of the donor and the recipient strains that were grown overnight in LB, washed and resuspended in either LB or M9 minimal medium. Equal amounts (10 μL) were mixed together and spotted onto LB or M9 agar plates that were incubated for 6 h at 37°C. pN3 conjugation frequency was determined as above. *, *P* < 0.05 (C) The fold change in the expression of six *tra* genes (*traK*, *traJ*, *traI*, *traG*, *traC,* and *traL*) in E. coli K1037 harboring pN3 grown in rich media (LB) and minimal media (M9) was determined using qRT-PCR. RNA was extracted from late logarithmic phase cultures grown at 37°C. The results show the mean value of 6–12 reactions from 2–4 independent RNA extractions, and the error bars indicate the SEM. One Sample *t* test against a theoretical value of 1.0 was used to determine statistical significance. ***, *P* < 0.001. (D) E. coli K1037 harboring pN3 and expressing a 2HA-tagged version of TraL and TraK were grown in rich (LB) and M9 minimal media at 37°C. Bacterial lysates were separated by SDS-PAGE followed by Western blotting using antibodies against hemagglutinin and the membranes were stained with Ponceau S as a loading control. Quantified bends densitometry is shown under the blots of TraK-2HA and TraL-2HA.

To analyze the possible effect of environmental cues on pN3 conjugation we further studied conjugation efficiency and the pattern of *tra* gene expression under various growth conditions. Conjugation experiments between E. coli K1037 harboring the pN3 plasmid and E. coli K-12 ORN172 that were performed in liquid LB broth and on solid LB agar demonstrated about 20-fold higher conjugation frequency on solid versus liquid medium (Fig. 4A). Moreover, mating experiments on solid LB and minimal medium M9 agar plates exhibited about 2.5-fold higher conjugation frequency on LB than on minimal medium plates (Fig. 4B).

Next, RNA was extracted from donor cultures that were grown to the late logarithmic phase in liquid LB and in M9 medium and was subjected to qRT-PCR. Interestingly, *traK*, *TraJ*, *traI*, *traG*, *traC*, and *traL* demonstrated 2- to 6-fold higher transcription levels in cultures grown in liquid M9 medium compared to growth in rich LB broth (Fig. 4C). In contrast, Western blotting against TraL-2HA and TraK-2HA showed elevated expression of TraL-2HA and TraK-2HA grown in LB relative to growth in M9 (Fig. 4D). These results indicated higher conjugation frequency of pN3 on solid versus liquid and in rich versus minimal medium, but also suggested differences in the regulation of *tra* genes between the transcriptional and the translational levels.

### The effect of temperature on pN3 conjugation.

**FIG 5:**
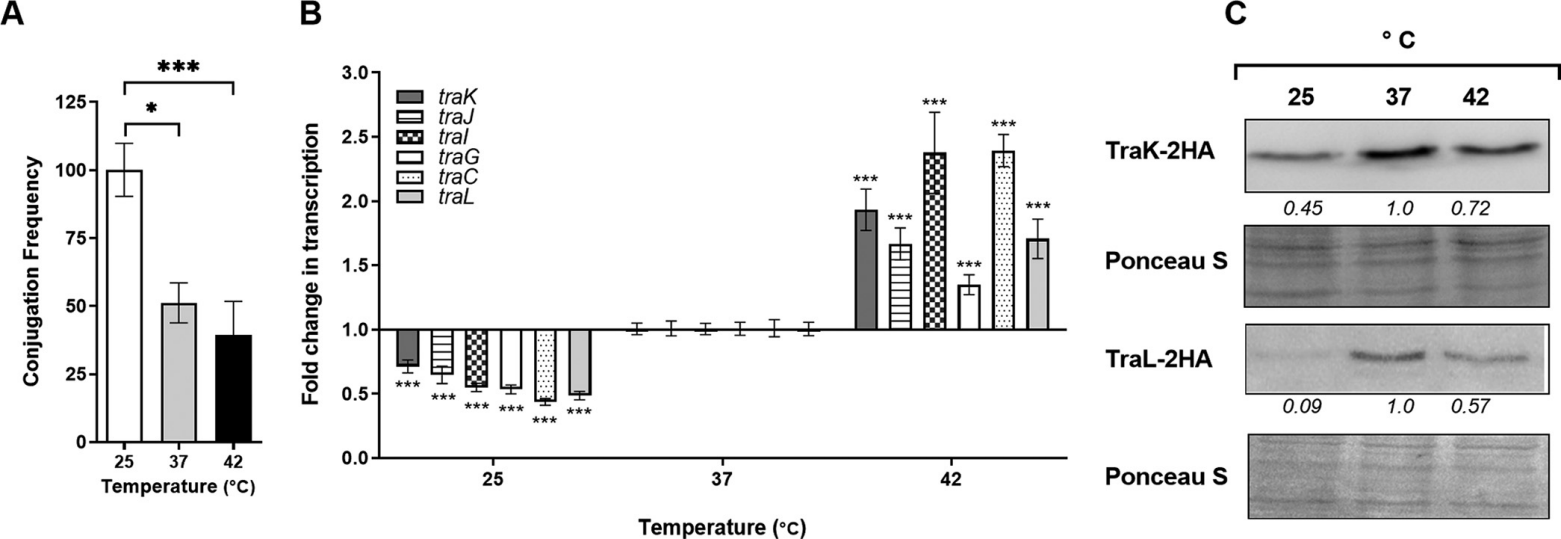
pN3 conjugation is affected by the ambient temperature. (A) Overnight grown cultures of the donor (E. coli K1037 harboring pN3 plasmid) and the recipient (E. coli K-12 ORN172) strains were mixed together in equal volumes and spotted onto LB agar plates that were incubated for 6 h at 25°C, 37°C, and 42°C. pN3 conjugation frequency was determined by the ratio between the obtained transconjugant CFU and the initial number of the donor CFU in each mating experiment. The chart shows the mean of three independent experiments with the SEM indicated by the error bars. One-way Anova with Dunnett test was used to determined statistical significance. *, *P* < 0.05; ***, *P* < 0.001. (B) Total RNA was extracted from E. coli K1037 harboring pN3 grown in LB to the mid logarithmic phase at 25°C, 37°C, and 42°C. qRT-PCR was applied to define the fold change in *traK*, *traJ*, *traI*, *traG*, *traC,* and *traL* transcription at 25°C and 42°C relative to the expression at 37°C. The results show the mean value of 6 reactions from two independent RNA extractions, and the error bars indicate the SEM. One Sample *t* test against a theoretical value of 1.0 was used to determine statistical significance. ***, *P* < 0.001. (C) E. coli K1037 harboring pN3 and expressing a 2HA-tagged version of TraL and TraK were grown in LB medium at 25°C, 37°C and 42°C. Bacterial lysates were separated by SDS-PAGE followed by Western blotting, using antibodies against hemagglutinin. The membranes were stained with Ponceau S for a loading control. Quantified bends densitometry is shown under the blots of TraK-2HA and TraL-2HA.

Temperature is an important environmental signal that basically affects all cellular functions. To manage unexpected temperature changes, all living cells constantly assess their ambient temperature using various sensing mechanisms, including regulatory proteins, alterations in membrane fluidity, and changes in DNA and RNA topology (43). Conjugation experiments conducted on LB agar plates that were incubated at 25°C, 37°C and 42°C, showed that the highest conjugation frequency of pN3 occurred at 25°C and decreased at higher temperatures (Fig. 5A). However, as in the case of the growth media, higher transcription of *tra* genes was in fact identified at 37°C and 42°C, in which lower conjugation frequency was observed, rather than at 25°C (Fig. 5B). In partial agreement with these results, Western blotting indicated that the highest expression level of TraK-2HA and TraL-2HA was found at 37°C, in comparison to their expression at 25 or 42°C (Fig. 5C). Taken together, our experiments suggest that in the case of pN3, there is no direct association between the expression level of *tra* genes and its conjugation frequency and that higher expression does not necessarily result in more frequent conjugation.

### The effect of osmolarity on pN3 conjugation.

**FIG 6:**
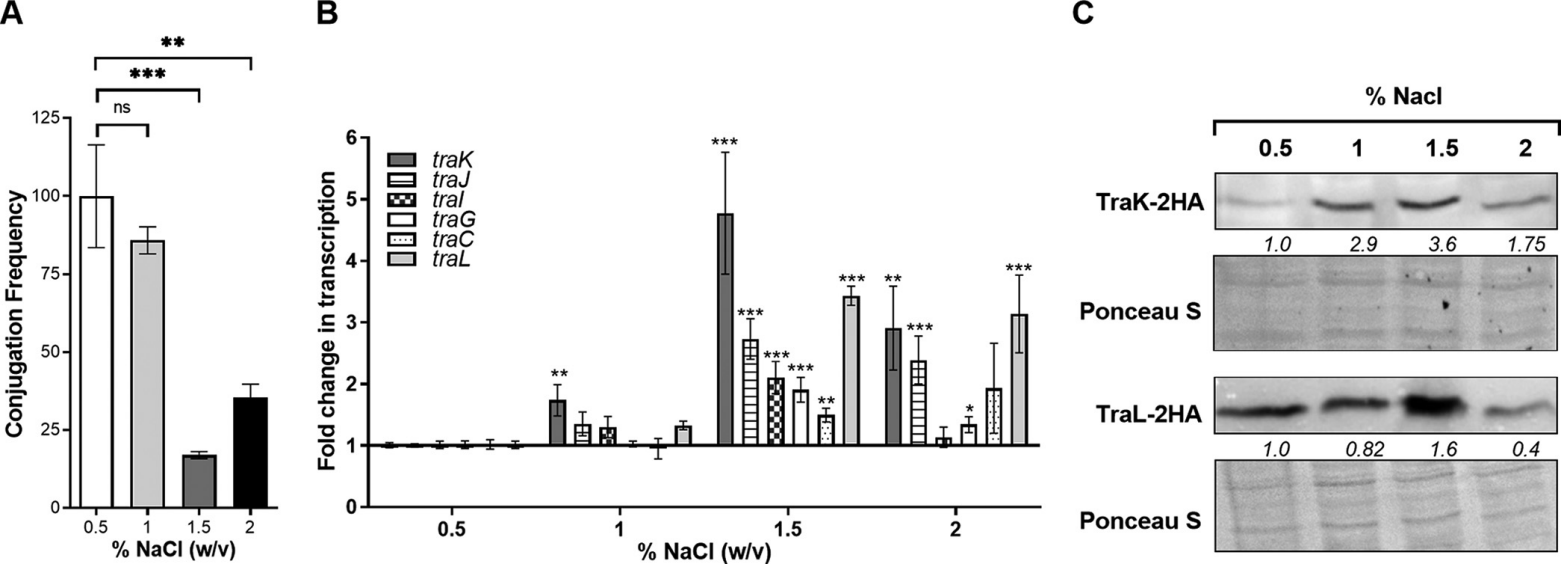
pN3 conjugation is affected by osmolarity. (A) Overnight grown cultures of the donor (E. coli K1037 harboring pN3 plasmid) and the recipient (E. coli K-12 ORN172) strains were washed and resuspended in fresh LB medium. Equal amounts (10 μL) of bacterial suspensions were mixed together and spotted onto LB agar plates supplemented with 0.5, 1, 1.5 and 2% (W/V) NaCl and incubated for 6 h at 37°C. pN3 conjugation frequency was determined by CFU enumeration of the obtained transconjugants (resistant to kanamycin and tetracycline) relative to the initial number of donor CFU. The bars show the mean of three independent experiments with the SEM indicated by the error bars. One-way Anova with Dunnett test was used to determined statistical significance. ns, not significant; **, *P* < 0.01; ***, *P* < 0.001. (B) The fold change in the expression of six *tra* genes (*traK*, *traJ*, *traI*, *traG*, *traC,* and *traL*) in E. coli K1037 harboring pN3 grown in LB medium supplemented with various concentrations of NaCl was determined using qRT-PCR. RNA was extracted from late logarithmic phase cultures grown under aerobic conditions at 37°C and the expression of the target genes was normalized to the expression of the stably expressed 16S rRNA gene. The chart bars show the mean value of 8 reactions from 2–3 independent RNA extractions, and the error bars indicate the SEM. One Sample *t* test against a theoretical value of 1.0 was used to determine statistical significance. *, *P* < 0.05; **, *P* < 0.01; ***, *P* < 0.001. (C) E. coli K1037 harboring pN3 and expressing a 2HA-tagged version of TraL and TraK were grown in LB media supplemented with 0.5, 1, 1.5, and 2% NaCl at 37°C. Whole cells bacterial lysates were separated by SDS-PAGE followed by Western blotting using monoclonal antibodies against hemagglutinin. The membranes were stained with Ponceau S and imaged for a loading control. Quantified bends densitometry is shown under the blots of TraK-2HA and TraL-2HA.

Bacteria can live and survive in a wide range of osmotic environments that range from highly hypotonic niches, such as freshwater reservoirs to high salinity environments. However, high osmolarity can cause stress to bacteria, due to the difficulty to take up water (44). To test the effect of osmolarity on bacterial conjugation, pN3 conjugation frequency from E. coli K1037 to E. coli K-12 ORN172 was studied in rich LB medium containing 0.5, 1, 1.5, and 2% NaCl. These experiments indicated that supplementing the growth medium with NaCl to concentrations of 1.5 or 2% significantly reduced the conjugation frequency of pN3 (Fig. 6A).

qRT-PCR, again showed an inverse relationship between the conjugation frequency and *tra* gene transcription, with the highest expression at 1.5% NaCl, where the conjugation frequency was the lowest (Fig. 6B). In agreement with the transcription data, Western blotting also indicated induction of TraK-2HA and TraL-2HA translation in cultures grown in LB supplemented with 1.5% NaCl (Fig. 6C). We concluded from these experiments that osmolarity affects pN3 conjugation, which decreases at NaCl concentrations of 1.5% and that unintuitively, under these conditions induced transcription and translation of at least some *tra* genes is observed.

### The effect of oxygen concentration on pN3 conjugation.

**FIG 7:**
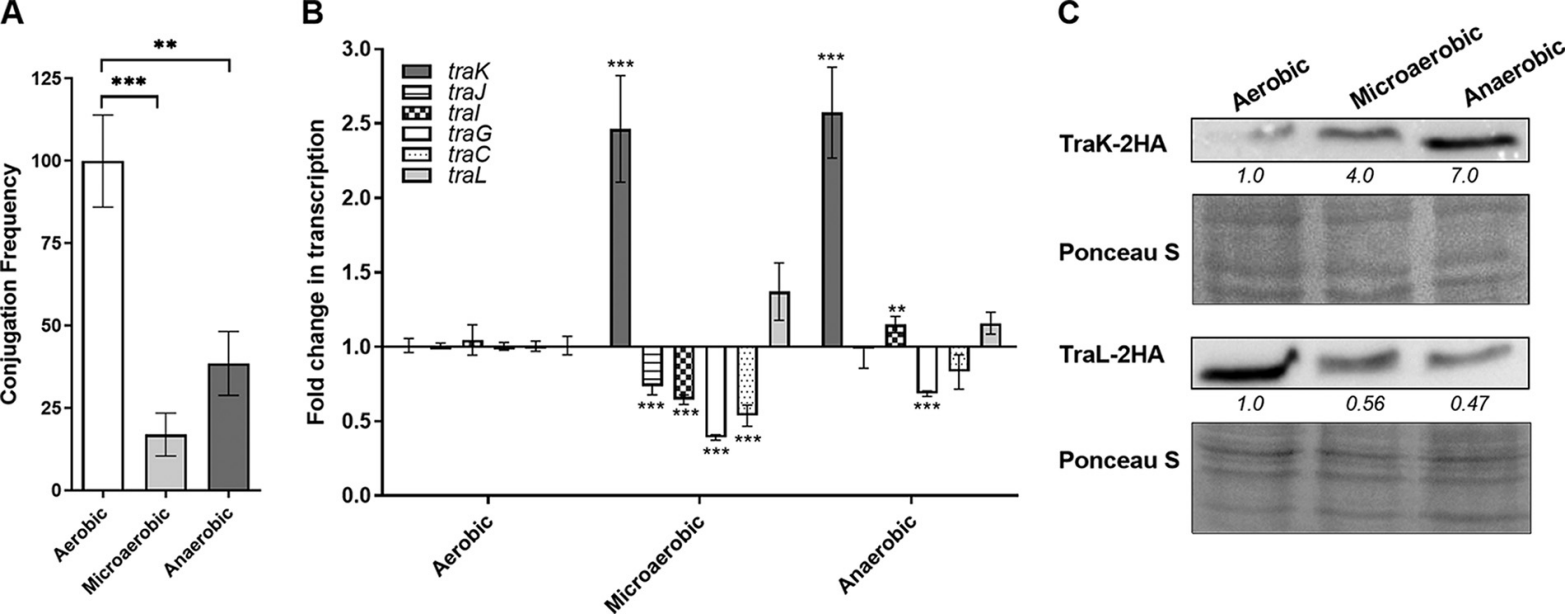
pN3 conjugation is affected by oxygen concentrations. (A) Overnight grown cultures of the donor (E. coli K1037 harboring pN3 plasmid) and the recipient (E. coli K-12 ORN172) strains were washed and resuspended in fresh LB medium. Equal amounts (10 μL) of bacterial suspensions were mixed together and spotted onto LB agar plates that were incubated for 6 h under aerobic, microaerobic and anaerobic conditions at 37°C. pN3 conjugation frequency was determined by CFU enumeration of the obtained transconjugants relative to the initial number of donor CFU in each mating experiment. The chart shows the mean of four independent experiments and the error bars represent the SEM. One-way Anova with Dunnett test was used to determined statistical significance. **, *P* < 0.01; ***, *P* < 0.001. (B) E. coli K1037 harboring pN3 cultures were grown in LB medium and incubated under aerobic (~5.9 mg/L O_2_), microaerobic (~3.9 mg/L O_2_) and anaerobic (~1.2 mg/L O_2_) conditions to the late logarithmic phase at 37°C. The fold change in the expression of *traK*, *traJ*, *traI*, *traG*, *traC,* and *traL* was determined using qRT-PCR and their expression was normalized to the expression of the stably expressed 16S rRNA gene. The chart bars show the mean value of 6–9 reactions from 2–3 independent RNA extractions, and the error bars indicate the SEM. One Sample *t* test against a theoretical value of 1.0 was used to determine statistical significance. (C) E. coli K1037 harboring pN3 and expressing a 2HA-tagged version of TraL and TraK were grown in LB media and incubated under aerobic (~5.9 mg/L O_2_), microaerobic (~3.9 mg/L O_2_) and anaerobic (~1.2 mg/L O_2_) conditions to the late logarithmic phase at 37°C. Whole cells bacterial lysates were separated on SDS-PAGE followed by Western blotting using monoclonal antibodies against hemagglutinin. The transferred membranes were stained with Ponceau S and imaged for a loading control. Quantified bends densitometry is shown under the blots of TraK-2HA and TraL-2HA.

Next, we studied pN3 conjugation under different oxygen concentrations. To this end, conjugation between donor and recipient strains was conducted on solid LB agar plates that were incubated under aerobic, microaerobic and anaerobic conditions. We found that pN3 conjugation frequency significantly decreased following growth at microaerobic and anaerobic concentrations (Fig. 7A).

Bacterial RNA that was extracted from the donor cultures grown in liquid broth to the late logarithmic phase under aerobic (~5.9 mg/L O_2_), microaerobic (~3.9 mg/L O_2_) and anaerobic (~1.2 mg/L O_2_) conditions was subjected to qRT-PCR analysis. Interestingly, here again we observed an inverse correlation between the conjugation frequency and the transcription of *traK* that increased by about 2-fold under microaerobic and anaerobic growth conditions (Fig. 7B). Concurringly, Western blotting against TraK-2HA also demonstrated elevated expression of TraK under microaerobic and anaerobic growth conditions (Fig. 7C), when the conjugation frequency of pN3 was lower. We concluded from these experiments that maximal conjugative transfer of pN3 occurs under aerobic conditions, however, at least *traK* expression is upregulated both at the transcriptional and translation level, under microaerobic and anaerobic conditions.

### Overexpression of *traL* or *traK* reduces pN3 conjugation.

**FIG 8:**
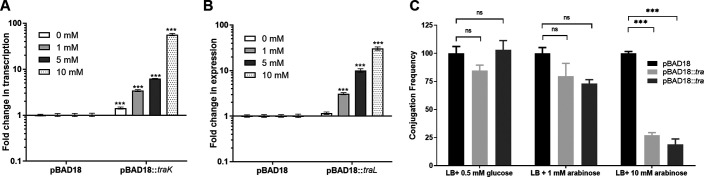
Induced expression of TraL or TraK reduces conjugation efficiency. E. coli K1037 harboring pN3 were transformed with pBAD18 (empty vector), pBAD18::*traK*, or pBAD18::*traL* and grown in LB supplemented with 1, 5 or 10 mM arabinose to the late logarithmic phase at 37°C. The fold change in the expression of *traK* (A) or *traL* (B) under arabinose induction was determined by qRT-PCR and normalized to the expression of the 16S rRNA gene. The chart bars show the mean value of 6 reactions from 2 independent RNA extractions, and the error bars indicate the SEM. One Sample *t* test against a theoretical value of 1.0 was used to determine statistical significance. ***, *P* < 0.001. (C) E. coli K1037 harboring pN3 and pBAD18, pBAD18::*traK*, or pBAD18::*traL* were grown for overnight in LB supplemented with 0.5 mM glucose and used as the donor strain. E. coli K-12 ORN172 that was used as the recipient strain was grown for overnight in LB. 10 μL from the donor and the recipient strains were mixed onto LB agar plates supplemented with 0.5 mM glucose, 1 mM arabinose, or 10 mM arabinose and incubated for 6 h at 37°C to conjugate. pN3 conjugation frequency was determined by CFU enumeration of the obtained transconjugants (resistant to kanamycin and tetracycline) relative to the initial number of donor CFU in each mating mix. The bars show the mean and SEM of three independent biological experiments. One-way Anova with Dunnett test was used to determined statistical significance. ns, nonsignificant; ***, *P* < 0.001.

The accumulating evidence that suggested under different growth conditions an inverse correlation between pN3 conjugation frequency and the expression of TraL and/or TraK was intriguing. We hypothesized that unsynchronized expression of *traL* or *traK* that is induced in response to certain environmental signals may negatively affect pN3 conjugation and reduce its frequency. To test this possibility experimentally, we cloned *traL* and *traK* from pN3 into pBAD18 under an arabinose inducible promoter, introduced these constructs into E. coli K1037 carrying pN3 and tested the expression of these genes as well as pN3 conjugation frequency following induction with arabinose. As illustrated in Fig. 8A and B, growing the bacteria in the presence of 1, 5, and 10 mM arabinose has led to induction of *traL* and *traK* transcription in a dose-dependent manner. Importantly, the induced strains also demonstrated a significant reduction in pN3 conjugation frequency that was dependent on the arabinose induction (Fig. 8A to C). An empty pBAD18 vector had no effect on the expression of *traL* nor *traK* and did not change the conjugation frequency of pN3 at any of the examined arabinose concentrations. We concluded from these results that induced expression of either *traL* or *traK* results in the reduction of pN3 conjugation frequency.

## DISCUSSION

Bacterial conjugation is a major genetic driver, enabling changes in complex phenotypes, including metabolic properties, symbiotic or parasitic lifestyles, pathogenicity, biofilm formation, resistance to heavy metals, and, most importantly form a clinical perspective, resistance to antibiotics (8). Therefore, conjugation is a powerful mechanism that shapes the evolution, diversification, and adaptation of bacterial communities. Conjugation was shown to be a ubiquitous phenomenon that occurs in bacterial populations occupying diverse ecological niches like soil, rhizosphere, phylloplane, aquatic environments, sewage, biofilms, as well as organs and tissues of animal hosts (45, 46). As such, various environmental factors, including temperature, pH, redox status, moisture, and the presence of pollutants, were shown to affect *tra* gene expression and the conjugation efficiency of several conjugative plasmids (8, 47, 48).

In Gram-negative bacteria, conjugation efficiency is typically high in derepressed donor cells. Usually, the conjugative apparatus is regulated by one main repressor such as the FinOP fertility inhibition system that keeps the conjugation genes in the default ‘OFF’ mode. After responding to an inducing signal, the conjugation genes are switched “ON,” by the activity of antirepressors or activators (19). Nonetheless, most of these studies were focused on the first discovered and extensively studied F or F-like plasmids (17, 49). It is now becoming apparent that there is a remarkable diversity among conjugative systems and in the way by which conjugation genes are regulated in different groups of conjugative plasmids (19, 47). Here, we chose to elucidate the conjugation activity and the regulation of pN3 transfer, an under studied conjugative system that we have used as a model to extend the knowledge about the regulation of non-F plasmid systems.

We showed that pN3-dependent conjugation and dissemination of antibiotic resistant genes via conjugation can occur in outer space and on the ground under microgravity-simulated conditions. These results suggest that conjugation, as a multistep process that involves complexes of >15 proteins composing the conjugative pili and the MPF can be assembled and function under low gravity conditions. To the best of our knowledge, this is the first evidence that bacterial conjugation can take place in outer space. This observation indicates that plasmid acquisition and subsequent, the development of antibiotic resistant or hyper-virulent strains due to conjugation are possible outside the earth's atmosphere. This information could be relevant for professional astronauts during missions in outer space, including in the international space station (ISS) and for space tourists who travel to space for recreational purposes and need to take into account the possibility of HGT among microbiota and environmental bacteria outside earth.

Moreover, we demonstrated that microgravity, elevated physiology-relevant temperatures, minimal growth medium, high osmolarity, and low concentration of oxygen reduce the pN3 conjugation frequency. Similar effects of temperature (50, 51) and anaerobiosis (50) were previously shown for IncP1 mercury resistance conjugative plasmids. Decrease of conjugation transfer at 37°C compared to 30°C was also demonstrated for the IncHI plasmid R27 (52), while lower frequencies of RP1 plasmid conjugation from Pseudomonas aeruginosa to E. coli was also observed at anaerobic versus aerobic conditions (53).

Conversely, these conditions may have a different effect on F or F-like plasmids. For example, the maximal conjugation occurrence of pN3 under aerobic conditions, and its reduced conjugation under microaerobic conditions contrasts with what was shown for the F-like plasmid pSLT, the virulence plasmid of S. enterica serovar Typhimurium. pSLT conjugation was shown to be repressed under aerobic conditions, but induced under microaerobiosis, in a manner that is depended on the ArcAB two-component regulatory system responsive to oxygen level (54). Similarly, while pN3 conjugation was found to be inhibited by high osmolarity, high-salt stress conditions were shown to increase the transfer frequency of F1 and Inc I1 plasmids (55). These results emphasize the diversity of conjugation regulation among Gram-negative bacteria and the fact that similar environmental stimuli differently affect the conjugation of distinct plasmids, possibly due to different genetic organization of the *tra* genes and ecological features of each plasmid. Indeed, some genetic features of pN3 are different from the genetic organization of the *tra* genes in F plasmids, including the location and the organization of the pJ and pY promoters that control the expression of the *tra* genes in F-plasmids (56, 57).

While current models suggest a direct correlation between *tra* gene transcription and conjugation frequency (8, 19, 47), our data point to a more complicated relationship. Interestingly, in pN3, during growth at rich versus minimal media, different temperatures, high osmolarity and microgravity we observed an inverse correlation between transcription of specific *tra* genes and conjugation. Moreover, we found that the induction of at least *traK* and *traL* can negatively affect the pN3 conjugation frequency in a dose-dependent manner. In pKM101 (21), which is the most studied IncN plasmid, TraL, was shown to be an VirB1 ortholog and found to partly complement the function of the VirB1 protein of A. tumefaciens (42). VirB1 functions as a lytic transglycosylase, providing a localized lysis of the peptidoglycan cell wall to allow insertion of the T4SS (58). Also in pKM101, TraK was recently shown to act as a DNA transfer and replication protein that binds at the *OriT* sequence as part of the relaxosome complex and is required to transfer of DNA and protein substrates through the pKM101-encoded T4SS, with possibly a few other functions (41). Since TraK is a predicted member of the Ribbon-Helix-Helix (RHH) family of DNA-binding proteins, which were originally defined as transcriptional repressors (59–61), it is tempting to speculate that TraK may also act as a transcriptional repressor, which negatively regulates the expression of other *tra* genes. Another possibility is that the lack of the FinOP and FinQ and FinW repression systems in the bacterial donor strain used here allows a constitutive expression level of the conjugative apparatus that is already sufficient for functional conjugation and that further induction of the *tra* genes leads to unsynchronized expression that impairs conjugation.

Differences between the effect of environmental cues on *tra* gene expression and conjugation frequency also highlight that the expression of *tra* genes is controlled by complex regulatory circuits, which involves joint activities of plasmid- and chromosome-encoded regulators coordinating expression at the transcriptional and posttranscriptional levels. Such complex regulation orchestrates conjugation in tandem with the plasmid ecology and the physiology of its carrier, in response to environmental conditions and interaction with other bacterial cells.

Sputter coating with iridium provides a stable and extremely thin conductive layer facilitating high resolution imaging of biological samples by scanning EM (28, 29). This preparation technique enabled us to visualize the fine ultrastructural details of the conjugative pili and the mating pair formation complexes in a “naked” E. coli surrogate strain, during cell-to-cell pili interactions between two pN3-positive bacteria. This phenomenon, known as phenocopy, was previously shown to promote E. coli biofilm formation (62). Scanning EM imaging indicated the simultaneous presence of both the conjugative pili that present as rigid 1–2.5 μm long fimbriae and the conjugative bridges that connect the cytoplasm of the mating cells. Previous studies have suggested that pilus morphology defines the ability of plasmids to transfer on solid or in liquid media. Rigid pili, like the ones found in Inc groups M, N, P, and W were shown to transfer better on solid media than in liquid environments (63). Indeed, studying the conjugative frequency of pN3 on solid versus liquid medium, has shown much higher frequency on solid LB agar plates than in liquid culture, in agreement with the observed morphology of the pN3 pili. While the structure of the pKM101-encoded T4SS was recently visualized by *in situ* cryo-electron tomography (64), to the best of our knowledge, these are the first published EM images of an IncN plasmid mating pair formation and intact conjugative pili structures.

In summary, we present visual, functional and expression characterization of the conjugative system encoded on pN3. We demonstrated functional conjugation between E. coli strains in outer space, outside earth’s atmosphere and showed the effect of various environmental conditions on conjugation frequency and *tra* gene expression, both at the transcriptional and the translational level. We showed complex regulatory circuits in pN3, with no direct correlation between the level of expression and conjugation efficiency, in a way that challenges previous models. Moreover, our results also suggest that optimal conjugation requires synchronized and orchestrated expression of *tra* genes and that transcriptional induction of either *traL* or *traK* results in impaired conjugation of pN3, in a dose-dependent manner. Collectively, these results highlight the diversity in conjugation regulation among distinct conjugation systems and the different ways they may be regulated in response to environmental signals. Future work is expected to shed more light on the mechanisms responsible for the integration of environmental cues and the regulatory cross talk between plasmid and chromosomal factors involved in these processes and how differences in the genetic organization of *tra* genes on different plasmids affect these regulatory circuits.

## MATERIALS AND METHODS

### Bacterial strains and culture conditions.

Bacterial strains and plasmids are listed in Tables S1. Bacterial cultures were routinely maintained in Luria-Bertani (LB) liquid medium or on LB agar plates. M9 medium with glucose (22 mM) was used as minimal medium. Microaerobic and anaerobic environments were produced using the CampyGen (Thermo Scientific) and the GasPak (BD Diagnostics) sachets gas generating systems, that were place in sealed containers without shaking. Media were supplemented with the appropriate antibiotics at the following concentrations: tetracycline 20 μg/mL, kanamycin 50 μg/mL, and ampicillin 100 μg/mL, when appropriate.

### Molecular biology.

All primers used in this study are listed in Tables S2. Oligonucleotides were purchased from IDT. Phusion Hot Start Flex DNA polymerase (New England BioLabs) was used for DNA amplification by PCR. For Western blot analyses, we first constructed a C-terminal 2HA tag for TraK and TraL. The genes *traK* and *traL* were PCR amplified from E. coli K1037 harboring pN3 as a template, with the primers 'traK 2HA FW' and 'traK 2HA RV' for *traK* amplification and 'traL 2HA FW' and 'traL 2HA RV' for *traL* amplification. Gel-purified fragments were cloned into pWSK29 containing a C-terminal two-hemagglutinin (2HA) encoded tag using SacI and XbaI restriction enzymes. For the arabinose-induced expression, *traK* and *traL* were amplified using the primers 'traK OE FW' and 'traK OE RV' for *traK* amplification and 'traL OE FW' and 'traL OE RV' for *traL* amplification. Gel-purified fragments were cloned into pBAD18 under araBAD promoter using SacI and HindIII. All ligation reactions were carried out using T4 DNA Ligase (New England BioLabs). Ligated plasmids were electroporated into E. coli MC1022 and purified plasmids were verified by sequencing and transformed by electroporation into E. coli K1037 harboring N3 plasmid.

### Mating experiments.

Agar mating method (65) was used to determine the conjugation frequency. A fresh colony from the donor strain E. coli K1037 harboring N3 plasmid (tetracycline resistance) and the recipient strains E. coli K-12 ORN172 (kanamycin resistance) or E. coli J-53 (rifampicin resistance) were grown overnight in liquid LB with the appropriate antibiotics. The next day, 1 mL of each culture was centrifuged for 2 min at 9500 g and washed with 100 μL of LB (without antibiotics). For the conjugation assay, 100 μL of the donor and the recipient cultures were mixed together in a test tube and 20 μL of the conjugation mix, or 10 μL from the donor suspension only were spotted onto an appropriate agar plate (LB or M9) and incubated for 6 h under interchanging conditions. Subsequently, the bacteria were scraped from the plate and resuspended in 1 mL saline (0.9% NaCl). Serial dilutions were made in saline and plated onto LB agar plates supplemented with tetracycline and kanamycin. The plates were incubated at 37°C overnight for transconjugants CFU count. Conjugation frequency was determined by the ratio between the number of obtained transconjugant CFU and the donor CFU.

To make sure that under these experimental conditions, tetracycline and kanamycin resistant E. coli are the result of a *bona fide* conjugation and cannot rise due to spontaneous mutations in the donor or the recipient strains, we have conducted 11 independent conjugation experiments and detected separately the frequencies of tetracycline and kanamycin resistant colonies during conjugation and in similar experiments that included the donor or the recipient cells only. As can be seen in Tables S3 and S4, we did not detect any donor or recipient colony that was tetracycline and kanamycin resistant in any of the ground control experiments, confirming that spontaneous mutations leading to such resistance profile is highly unlikely, under these experimental conditions, even during a prolonged incubation of 40 days.

### Conjugation under microgravity-simulated conditions.

Overnight cultures of the donor (E. coli K1037 harboring N3 plasmid) and the recipient (E. coli K-12 ORN172) strains were grown in LB supplemented with tetracycline and kanamycin, respectively, at 37°C. The next day, the donor and the recipient were mixed in 1:1 ratio, and 10 mL of the mixture were loaded onto the RWV 10 mL disposable vessels (Synthecon). One plate was placed on the RWV bioreactor, and the control plate was placed on a standard roller drum. Both instruments were incubated at 37°C and operated at a rotation speed of 43 rpm for 6 h.

### Scanning electron microscopy.

E. coli K-12 ORN172 bacteria harboring pN3 were grown on LB agar plates supplemented with tetracycline and kanamycin. The next day, bacteria were gently scraped off using a bacterial inoculation loop and lightly resuspended in 500 μL saline. 100 μL of the bacterial suspension were applied onto fibronectin-coated 13 mm round “Deckglaser” coverslips and incubated for 1 h at room temperature. Following the incubation, coverslips were transferred into 24-well plates, 1 coverslip per well, filled with 1 mL fixation buffer containing 80 mM PIPES NaOH, pH 6.8, 1 mM MgCl_2_, 150 mM sucrose, 2% paraformaldehyde (Electron Microscopy Sciences, [EMS]), 2% glutaraldehyde (EMS) for 20 min at room temperature. Samples were then washed twice for 2 min with 1 mL of 0.1 M sodium cacodylate buffer pH 7.4 (EMS). Secondary fixation was performed with 2% osmium tetroxide in 0.1 M sodium cacodylate for 10 min. The samples were washed twice for 2 min each in double-distilled water (DDW) and then dehydrated (2 min, twice for each step) under a series of ethanol concentrations (7.5%, 15%, 30%, 50%, 70%, 90%, 95%, and 100%). Next, samples underwent critical-point drying using a K850 critical-point dryer (Quorum Technologies). Coating was performed with ~1 nm of iridium using a Q150T coater (Quorum Technologies). Samples were imaged in a *Merlin* scanning electron microscope (Zeiss).

### Quantitative real-time PCR analysis.

E. coli K1037 harboring the pN3 plasmid were grown overnight in LB at 37°C. The next day the bacteria were subcultured (1:100) and grown under the mentioned set of conditions. RNA was extracted from 500 μL of culture using the Qiagen RNA protect Bacteria Reagent and RNeasy minikit according to the manufacturer's instructions, including an on column DNase digest, using the RNase free DNase set (Qiagen). The quantity and quality of the extracted RNA were determined by Nanodrop 2000c (Thermo Fisher Scientific). To diminish any genomic DNA contamination, RNA was retreated with an RNase-free DNase (Qiagen). cDNA was synthesized using qScript cDNA Synthesis kit (Quanta-bio) in a T100 thermal cycler by (Bio-Rad Laboratories). Real-Time PCRs were performed in a StepOne Real-Time PCR instrument (Applied Biosystems). Each reaction was carried out in a total volume of 20 μL in a 96-well optical reaction plate (Applied Biosystems). Melting curve analysis verified that each reaction contained a single PCR product. The housekeeping *16S* rRNA gene, was used as the endogenous normalization control. The ΔCt values were calculated by determining the difference in threshold values for *16S* rRNA. Calculation of ΔΔCt involved the subtraction of the normalized ΔCt value of the gene of interest at the control experiment from the normalized ΔCt value of this gene under the tested condition. Fold-differences in gene expression were calculated as 2^−ΔΔCT^.

### Western blotting.

Overnight cultures grown in LB at 37°C were diluted 1:100 and subcultured for 3–4 h under the appropriate conditions. The cultures were OD_600_ normalized, centrifuged, and the pellets were resuspended in 100 μL 1×sodium dodecyl sulfate-polyacrylamide gel electrophoresis (SDS-PAGE) sample buffer (0.15 M Tris-HCl pH 6.8, 10% Mercaptoethanol, 1.2% SDS, 30% Glycerol, 0.04% Bromophenol blue). Boiled samples were separated on 12% SDS-PAGE and transferred to a polyvinylidene fluoride (PVDF) membrane (Bio-Rad Laboratories) as we previously described (66). Blots were probed with anti-HA tag antibody (Abcam; ab18181, diluted 1: 1,000). Goat anti-mouse antibody conjugated to horseradish peroxidase (Abcam; ab6789, diluted 1: 5,000) was used as a secondary antibody, followed by detection with enhanced chemiluminescence (ECL) reagents (Bio-Rad Laboratories). For a loading control, membranes were stained with Ponceau S solution (Sigma-Aldrich) and destained with dH_2_0. All blots were imaged using the Fusion solo X system (Vilber). Bends densitometry was determined by Image J as explained in (67).

### Bacterial conjugation in outer space.

The on-orbit experiment was conducted using the remote-controlled lab-on-chip microgravity platform SPmg2Lab (SpacePharma; Fig. S2C) that was placed inside a pressurized atmospheric box and installed onto the nanosatellite DIDO-3 (Fig. S2A-B). The lab-on-chip platform contained the fluid handling system, which is based on spring-operated flexible liquid reservoirs (plungers), interim mixing chambers (main chambers) and an agitated observation chamber visible by the optical system. The microgravity lab is also equipped with a manifold, which directs the fluid flow, a light source and a spectrometer with a wavelength range of 420 to 750 nm (Ocean Insight), positioned above the observation chamber.

Before the launch, a fresh colony from the donor strain E. coli K1037 harboring N3 plasmid (tetracycline resistance) and the recipient strain E. coli K-12 ORN172 (kanamycin resistance) were grown for overnight in LB with the appropriate antibiotics. The next day, bacteria were diluted to OD_600_ 0.5 in saline and loaded in two separate 1 mL and 0.5 mL chambers in the plunger unit. On orbit, 520 μL of the recipient culture were mixed with 680 μL of the donor in the observation chamber to achieve a final 1:1 mixture of donor and recipient strains. In addition, 600 μL of 3× LB broth was added to the observation chamber to a final concentration of 1× LB medium. Bacteria were grown for18 days in space in an orbit of about 530 km above earth, allowing conjugation to occur. After 18 days, 120 μL of 10 × LB with kanamycin and tetracycline was added to reach a final concentration of 1× LB with 20 μg/mL Tc and 50 μg/mL Km. Under these conditions only the transconjugant bacteria can grow and multiply. Spectrophotometer reading at 600 nm was used to determine the optical intensity of the culture at real time. These data were transmitted between 3 to 4 times a day to a SpacePharma ground station in Switzerland.
