# Supplementary material for: The Effect of Outer Space and Other Environmental Cues on Bacterial Conjugation
Source: Microbiol Spectr. 2023 Mar 30;11(3):e03688-22. doi: 10.1128/spectrum.03688-22 (PMC10269834; doi:10.1128/spectrum.03688-22)
Supplement: Supplemental file 1 — Supplemental material. Download spectrum.03688-22-s0001.pdf, PDF file, 0.5 MB [file spectrum.03688-22-s0001.pdf]

Fig. S1. Scanning EM imaging of the recipient strain. Scanning electron microscope was performed as in Figure 2 to image *E. coli* K-12 ORN172 that was used as the recipient (surrogate) strain for pN3 conjugation. The green arrows indicate the flagella of this bacterium and highlight their distinct morphology, that differs from that of the conjugative pili of pN3 expressing cells, as shown in Fig. 2.

Fig. S2. The DIDO-3 nanosatellite and the SPmg2Lab microgravity platform. (A) The DIDO-3 nanosatellite consists of three CubeSat units, one for the service module (left side) and two for the actual microgravity laboratory (SPmgLab2, right side) with dimensions of 35 × 10 × 10 cm and total weight of 5.6 Kg. The service module comprises an on-board computer (OBC), battery package, and professional circuit design (PCD) electronics. **(B)** The assembled DIDO-3 with deployable solar panels is shown. **(C)** The SPmgLab2 consists of a closed pressurized metallic atmospheric box comprising two plunger unit cassettes with fluid reservoirs, a dispensing fluid unit, a manifold which directs the fluid flow, an observation chamber, and a light source which is placed under it. Additionally, outside the atmospheric box, the lab includes an optic box with a camera, light microscope, and a spectrometer. The total weight of the SPmgLab2 is 2.5 Kg.

Table S1: Bacterial strains and plasmids used in this study:

**Strain**	**Description**	**Source or reference**
E. coli ORN 172	*E. coli* K-12 ORN12 *thr*-l *leuB thi*-1 Δ(*argF*-lac) U169 *xyl*-7 *ara*-13 *mtl*-2 *gal*-6 *rpsL tonA2 supE44* Δ(*fimBEACDFGH*)::kan *pilG1*	PMID: 8097517
E. coli J53	*E. coli* J53 F^- proA metF (Lac +)	PMID: 130378

E. coli K1037/ pN3	*E. coli* K1037 / pN3	PMID: 22475035
ORN172 /pN3	*E. coli* K-12 ORN172 / pN3	This study
MC1022	*E. coli* K-12 MC1022	PMID: 6997493
**Plasmid name**	**Description**	**Source**
pN3	incN plasmid N3	PMID: 22475035
pRP4	incP-α plasmid RP4	PMID: 5329290
pRK2	incP plasmid RK2	PMID: 8014987
pCVM	incI plasmid CVM	PMID: 19648374
pRL27	incQ plasmid RL27	PMID: 8693022
pESI	pESI plasmid	PMID: 32145019
pWSK29-2HA tag	2HA tag cloned into pWSK29	Lab collection
pWSK29-2HA::*traK*	*traK* cloned into pWSK29-2HA	This study
pWSK29-2HA::*traL*	*traL* cloned into pWSK29-2HA	This study
pBAD18	Arabinose-inducible expression plasmid	PMID: 7608087
pBAD18::*traK*	*traK* cloned into pBAD18	This study
pBAD18::*traL*	*traL* cloned into pBAD18	This study

Table S2: Primers used in this study:

**Primer name**	**Sequence (5' to 3')***	**Purpose**
traG RT F	GGGATCAGGGGCGTTATCTG	RT-PCR of *traG*
traG RT R	CTGAAAATCCCCTCGCTGGT	
traC RT F	GATCTACAGCGACGCCATGA	RT-PCR of *traC*
traC RTR	CGCCTTTTTCAGCCAGCTTT	
traL RTF	CTGGGTCTTTCGGTTGACGA	RT-PCR of *traL*
traL RTR	GTGAGTGAGCCGGTGTTGTA	
traK RTF	CGGGAGTCCGGGTTGAAAAT	RT-PCR of *traK*
traK RTR	TACGCCAGATAGCGTTTCGG	
traI RT F	CGCGACAGCCGTTATTTCAG	RT-PCR of *traI*
traI RT R	AACCGTCAATTTGTTGCCCG	
traJ RT F	ATCCGATCCTCAGAAGCCCT	RT-PCR of *traJ*
traJ RT R	TTGAACTTAGCGACGGGCAT	
16s rRNA RT F	GGTTAAGTCCCGCAACGAG	RT-PCR of 16S rRNA
16s rRNA RT R	CTTCTCTTTGTATGCGCCATTG	
traL 2HA FW	TTTTGAGCTCTCGTTTTTCTGATTTCAGGGGAA	Cloning of *traL* into pWSK29-2HA
traL 2HA RV	AAAATCTAGATTCCCCCTTCGCTGTTTCCT	
traK 2HA FW	GCTGGAGCTCAGTACCCTCATTTAGAATGATGTA	Cloning of *traK* into pWSK29-2HA
traK 2HA RV	AAAATCTAGA GCGCTCCTTTTTTGGTTCCC	
traK OE FW	TACCGAGCTC ATGCCAATAATAACCGCAAAAG	Cloning of *traK* into pBAD18
traK OE RV	TTTTCTCTCATCCGCCAAAACAGCCAAGCTT	
traL OE FW	TACCGAGCTC ATGAGTAAACATCCAAAACTCC	Cloning of *traL* into pBAD18
traL OE RV	AGCCAAGCTT TCATTCCCCCTTCGCTGT	

* Nucleotide sequence for restriction enzyme sites added to the primers are underlined.

Table S3: The frequency of transconjugants, donor and recipient strains resistant to tetracycline and kanamycin. A fresh colony from the donor strain *E. coli* K1037 harboring N3 plasmid (tetracycline resistance) and the recipient strains *E. coli* K-12 ORN172 (kanamycin resistance) were grown overnight in liquid LB with the appropriate antibiotics. The next day, 1 ml of each culture was centrifuged for 2 min at 9500 g and washed with 100 µl of fresh LB (without antibiotics). For the conjugation assay, 100 µl of the donor and the recipient cultures were mixed together in a test tube and 20 µl of the conjugation mix, or 20 µl from the donor or the recipient cells only were spotted onto an LB agar plate and incubated for 6 h at 37˚C. Subsequently, the bacteria were scraped from the plate and resuspended in 1 ml saline. Serial dilutions were made in saline and plated onto LB agar plates supplemented with tetracycline, kanamycin or tetracycline and kanamycin together. The plates were incubated at 37 °C overnight for CFUs count. The frequency of the transconjugants (number of transconjugant CFUs per donor CFUs) as well as the CFU/ ml count of the donor and the recipient strains is shown for 11 independent experiments. ND, not detected (i.e. no colonies were observed on the selective plates).

Experiment number	Frequency of Tet^R kan^R obtained for trans-conjugants	CFU count of Tet^R obtained for donor cells (*E. coli* K1037/pN3	CFU count of Tet^R kan^R obtained for donor cells *(E. coli* K1037/pN3	CFU count of kan^R obtained for recipient cells (*E. coli* K-12 ORN172)	CFU count of Tet^R kan^R obtained for recipient cells (*E. coli* K-12 ORN172)
1	2.67E-03	1.08E13	ND	2.78E+11	ND
2	6.94E-05	3.20E14	ND	3.50E+11	ND
3	4.49E-05	5.20E14	ND	1.40E+11	ND
4	1.52E-04	1.10E14	ND	2.40E+11	ND
5	1.21E-04	2.30E14	ND	2.00E+11	ND
6	4.00E-05	5.00E14	ND	1.50E+11	ND
7	4.04E-02	2.2E11	ND	1.1E+11	ND
8	1.06E-02	2.1E11	ND	2.7E+11	ND
9	7.07E-02	2.2E11	ND	3.5E+11	ND
10	8.55E-03	2.6E11	ND	2.9E+11	ND
11	7.94E-03	2.8E11	ND	2.2E+11	ND

Spontaneous rise of tetracycline and kanamycin resistance in the donor and the recipient strains is extremely infrequent

To further account that the growth of tetracycline and kanamycin resistant population in the conjugation experiments is not the result of spontaneous mutations in the donor or the recipient strains, we have conducted the following control experiment on the ground: *E. coli* K1037 harboring pN3 (donor strain) and *E. coli* K-12 ORN172 (recipient strain) were grown for overnight in LB medium and diluted in saline (0.7% NaCl) to an OD_{600} of 0.5, to achieve a similar bacterial density as the space cultures. Eight independent cultures of 1.2 ml of the donor and the recipient strains were placed in sterile 2 ml test tubes. Similarly, eight cultures that contained 600 µl of the donor and 600 µl of the recipient strains were also mixed in 2 ml test tubes and all of these 24 tubes were incubated at 22.2°C, to meet the average temperature of the space experiment. As was conducted in the space experiment, at day 18 post inoculum, 120 µl of fresh 10 × LB supplemented with kanamycin and tetracycline was added to each tube to reach a final concentration of 20 µg/ml tetracycline and 50 µg/ml kanamycin. At day 19 and 40, post inoculum, four donor cultures were serially deluded and plated on LB agar plates supplemented with tetracycline to determine CFUs count and on plates supplemented with tetracycline and kanamycin to examine possible rise of spontaneous double resistance phenotype. Similarly, recipient cultures were plated onto LB agar plates supplemented with kanamycin, and on selective plates supplemented with kanamycin and tetracycline. As shown in Table S4, under these experimental conditions, we did not detect any recipient or donor colony that has acquired a spontaneous resistance to tetracycline and kanamycin, while 1.32-4.62 X 10^5 and 1.0-2.03 X 10^7 double resistant CFUs were counted in the conjugation experiments at days

19 and 40, respectively. Therefore, we concluded from these control experiments presenting at Tables S3 and S4 that rising of a spontaneous resistance to tetracycline and kanamycin in the donor or the recipient strains is extremely infrequent and was undetected under these experimental conditions.

Table S4: The frequency of transconjugants, donor and recipient strains resistant to tetracycline and kanamycin following 19 and 40 days incubation.

The CFU count of kanamycin (Kan), and tetracycline plus Kanamycin (Tet+Kan) resistant recipient strain (R1 to R4), the CFU count of tetracycline (Tet), and tetracycline plus Kanamycin (Tet+Kan) resistant donor strain (D1 to D4), and the CFU count of transconjugants resistant to Tet+Kan (C1 to C4) is shown together with the dilution factor that was used to count the colonies (dilution). ND, not detected (i.e. no colonies were observed on the selective plates).

culture	19 days post inoculum						40 days post inoculum					
	Tet	dilution	Kan	dilution	Tet+kan	dilution	Tet	dilution	Kan	dilution	Tet+kan	dilution
R1			1.17E+09	10E-6	ND	undiluted			9.90E+07	10E-5	ND	undiluted
R2			1.08E+09	10E-6	ND	undiluted			6.47E+07	10E-5	ND	undiluted
R3			1.27E+09	10E-6	ND	undiluted			1.81E+08	10E-5	ND	undiluted
R4			1.45E+09	10E-6	ND	undiluted			1.76E+08	10E-5	ND	undiluted
D1	3.43E+08	10E-6			ND	undiluted	1.21E+07	10E-5			ND	undiluted
D2	2.38E+08	10E-6			ND	undiluted	1.04E+07	10E-5			ND	undiluted
D3	2.64E+08	10E-6			ND	undiluted	5.02E+06	10E-5			ND	undiluted
D4	3.17E+08	10E-6			ND	undiluted	2.55E+07	10E-5			ND	undiluted
C1					2.24E+05	10E-3					2.01E+07	10E-4
C2					1.32E+05	10E-3					1.00E+07	10E-4
C3					1.45E+05	10E-3					2.03E+07	10E-4
C4					4.62E+05	10E-3					1.97E+07	10E-4

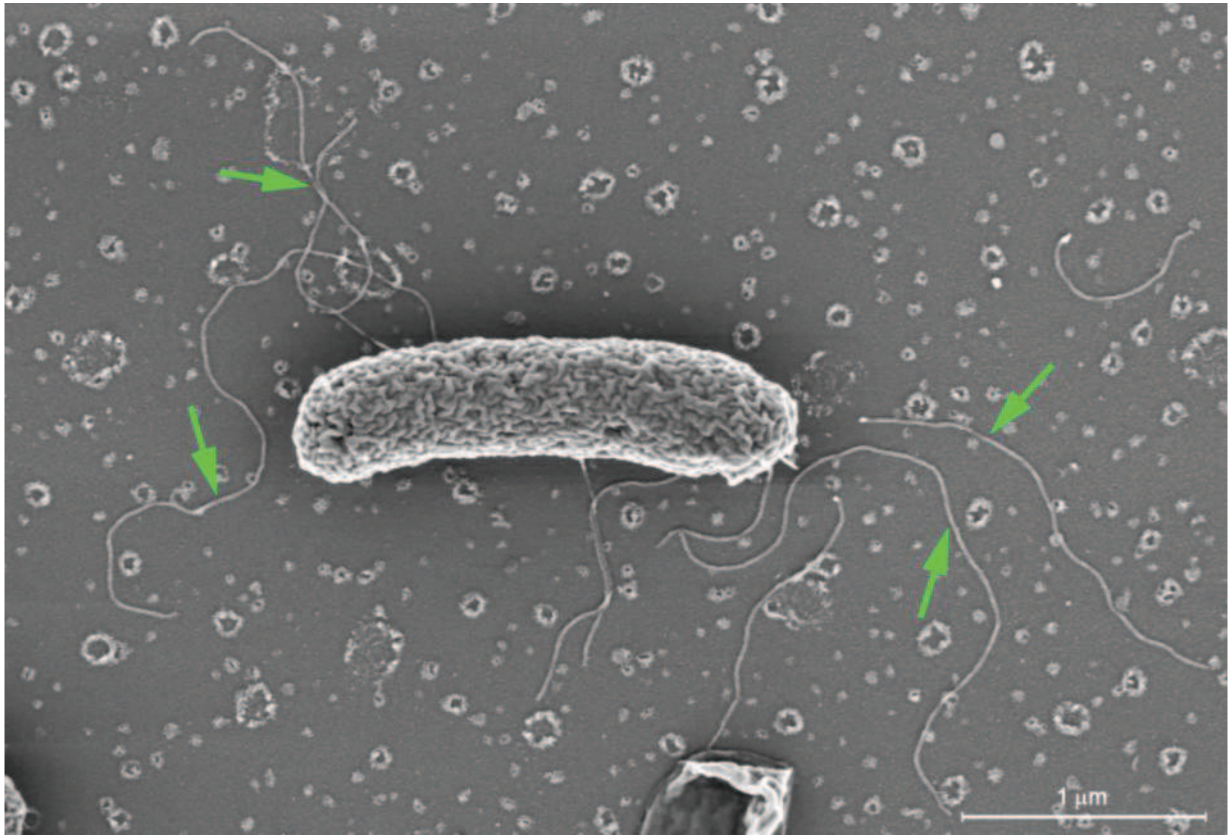

Fig. S1

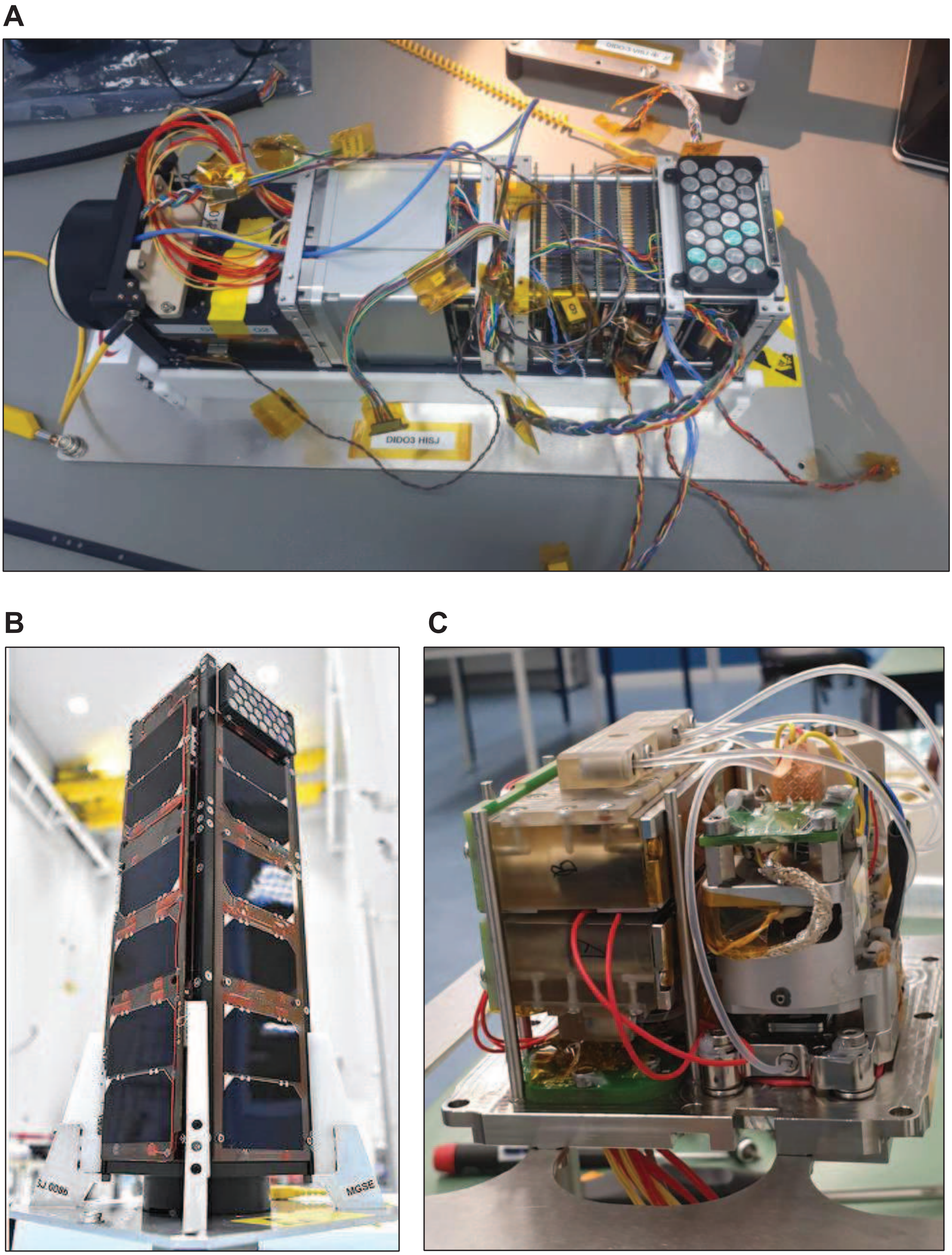

Fig. S2
